# Challenges and enablers of collaborative working in the voluntary, community, social, faith and enterprise sector: impact, effectiveness, and sustainability

**DOI:** 10.3389/fpubh.2026.1763039

**Published:** 2026-05-11

**Authors:** Britzer Paul Vincent, Nasreen Ali, Chimeme Egbutah, Karen Perkins

**Affiliations:** 1 Faculty of Health and Social Sciences, Institute for Health Research, University of Bedfordshire, Luton, United Kingdom; 2 Public Health, Luton Borough Council, Luton, United Kingdom; 3 Bedfordshire & Luton Community Foundation, Silsoe, United Kingdom

**Keywords:** collaboration, effectiveness, evaluation, impact, mixed-method approach, sustainability, VCSFE

## Abstract

**Introduction:**

Voluntary, Community, Social, and Faith Enterprise (VCSFE) organisations play a vital role in supporting local health and care needs, particularly among underserved communities. However, they can often operate in isolation, making it difficult for vulnerable populations to access coordinated support. Luton Public Health led a pilot called the Collaboration Fund that encouraged joint working among selected VCSFE organisations, delivering services that tackle health inequalities. The evaluation of the Collaboration Fund assessed the impact, effectiveness, and sustainability of this collaborative approach in addressing system approach to improving health outcomes.

**Methods:**

A parallel mixed-methods design was adopted to evaluate this collaborative approach among VCSFE organisations funded by the Collaboration Fund in Luton. Impact was assessed using quantitative data from five lead organisations. Effectiveness and sustainability were explored by conducting 13 qualitative interviews with project leads and managers. Descriptive statistics and geospatial mapping were used for quantitative analysis, and qualitative data were thematically analysed using Framework Analysis in NVivo v.20.

**Findings:**

The impact findings highlighted that 7,177 service users accessed services, mainly by women and those living in Luton’s most deprived wards. The effectiveness of working collaboratively improved service delivery through shared resources, co-designed services, and increased community trust and engagement. However, challenges included overlapping roles, limited communication, and a lack of diverse staff among a few of the organisations (One lead organisation and their three partner organisations). Sustainability was impeded by funding constraints, with stakeholders recommending stronger networks, shared outreach platforms, and improved visibility of the local VCSFE services to maintain and enhance collaboration.

**Conclusion:**

The Collaboration Fund enhanced inclusive, community-based services through strong partnerships, multilingual outreach, and creative engagement, improving access and reducing isolation among marginalised groups. Collaboration was most effective when organisations with strong community representation partnered with less diverse ones. Sustaining progress requires continued investment, resource sharing, and strategic support to deliver collaboration and address inequalities.

## Introduction

Voluntary, Community, Social, and Faith Enterprise (VCSFE) sectors in the United Kingdom (UK) play a crucial role in delivering health and care services (1). A primary objective of VCSFEs is to address health inequalities by supporting diverse populations, especially those most vulnerable, to enhance health and care outcomes (1). They are increasingly acknowledged as local experts and advocates, possessing valuable insights into community health needs and the skills to engage with underserved groups effectively. This aligns with the growing emphasis on population health management and inclusive approaches within integrated care frameworks (1).

The VCSFE sector in the UK is highly diverse, encompassing a wide range of organisations including grassroots community groups, faith-based organisations, social enterprises, and large voluntary bodies, each serving different populations and needs (2). This diversity allows the sector to engage effectively with varied communities, particularly those that are underserved or marginalised, offering culturally sensitive and tailored support (3). Moreover, the sector’s varied organisational forms and missions contribute to its flexibility and capacity to innovate within health and social care systems (4). Research has suggested that collaboration among such sectors improves the quality of care, access to care, and health outcomes (5).

The Health and Care Act (2022) established Integrated Care Systems (ICSs) as statutory partnerships aiming to improve population health, reduce inequalities, and enhance the efficiency of health and care services (6). The ICSs consist of Integrated Care Boards (ICBs), which are responsible for planning healthcare, and Integrated Care Partnerships (ICPs), comprising NHS organisations, local authorities, and the voluntary sector, to develop long-term strategies for improved health and wellbeing (7). Although not mandated by law, Place-Based Partnerships (PBPs) operate within larger ICSs, bringing together the NHS, local government, and the voluntary, community, faith, and social enterprise (VCFSE) sector to design integrated services tailored to local community needs (7–9). These structures collectively seek to provide more coordinated, inclusive, and effective health and care services across geographical regions (6–9).

The NHS 10-year plan has proposed 3 radical shifts in which the first one is ‘hospital to community’ (10). This new strategy aims to bring healthcare closer to communities through Neighbourhood Health Centres and strong partnerships with the VCSFE sector. By integrating local, digital, and home-based care, it will make services more accessible and person-centred. Together, communities and VCSE partners will help deliver preventative, continuous, and inclusive care for all (4). This puts the VCSFE sectors to potentially support the integrated service approach outlined in the new NHS 10-year plan, enabling outreach to the community and offering personalised, out-of-hospital care that promotes independence and reduces pressure on acute services (11, 12). While health and care provision are essential, there is broad recognition that the majority of factors influencing population health lie beyond the healthcare system itself. Therefore, this highlights the importance of VCSFE sectors in addressing the wider social determinants of health, which often fall beyond the remit of healthcare itself (13).

The VCSFE sector offers distinct advantages over core NHS healthcare services in engaging with communities, particularly marginalised and underserved populations (14). Unlike statutory services, VCSFE organisations often possess deep-rooted trust, cultural understanding, and longstanding relationships within local communities, enabling them to access groups that may be hesitant to engage with formal healthcare systems (15, 16). Their grassroots presence and flexibility enable person-centred, holistic support that addresses not only clinical needs but also the social determinants of health, such as housing, food security, and social isolation (17). Moreover, these organisations are adept at co-producing services with service users, fostering greater responsiveness and empowerment (14–16). In contrast, the NHS, while comprehensive and highly specialised, often faces challenges related to accessibility, bureaucracy, and uniform service models that may not fully reflect the diverse needs of local populations (18).

This evaluation focuses on the Collaboration Fund that evolved as a strategic initiative building upon the initial Contain Outbreak Management Fund (COMF) and the subsequent Community Recovery Fund (CRF), both established to address the immediate impacts of COVID-19 on marginalised communities in Luton (19, 20). The COMF was allocated to the Bedfordshire and Luton Community Foundation (BLCF), which established the Community Recovery Fund (CRF) to support organisations directly serving marginalised groups in post-COVID-19 recovery work (19–21). The CRF targeted organisations assisting vulnerable groups during post-pandemic recovery, focusing on mental health, social isolation, and disproportionately affected populations such as ethnic minorities and women (19, 20).

Building on lessons learned from previous projects involving the VCSFE sector and recognising the need for sustained and coordinated action, the Collaboration Fund was established to strengthen partnerships and promote a more integrated and collaborative model of working across the sector, operating between January 2023 and June 2024. Historically, many VSCFE organisations have operated largely in isolation, with limited coordination or connection between services. The Collaboration Fund therefore, sought to test and support a model that brings together organisations from across the VSCFE sector to work collaboratively, enabling them to pool expertise, resources, and networks. The overarching aim of this initiative was to enhance coordination between services and provide a more joined-up and responsive approach to meeting the diverse needs of service users. By fostering collaboration rather than fragmented service provision, the fund aimed to improve access to support, reduce duplication of efforts, and ultimately contribute to more effective and sustainable service delivery within the sector.

The Institute for Health Research (IHR) at the University of Bedfordshire was commissioned to undertake an evaluation of the services funded through the Collaboration Fund during the funding period from February 2024 to November 2024 (19, 20). The purpose of the evaluation was to examine the impact in terms of the reach of the funded services, the effectiveness of the partnership arrangements among organisations within the VSCFE sector, and the sustainability of continuing this collaborative model of working beyond the funding period.

## Methods

### Ethics

This paper is based on an evaluation approved by the Institute for Health Research Ethics Committee (IHREC1034), University of Bedfordshire, United Kingdom. Written and verbal consents were sought from all the participants.

### Evaluation design

The evaluation design followed a parallel mixed-methods approach (22), utilising both quantitative and qualitative data to examine the impact in terms of the reach of the funded services, the effectiveness in terms of the partnership arrangements among organisations within the VSCFE sector, and the sustainability in terms of continuing this collaborative model of working beyond the funding period (19, 20). This approach allows for the integration of numerical trends with in-depth contextual insights, enhancing the validity and richness of the findings (22). The impact in terms of the reach of the services of the Collaboration Fund is represented using quantitative data, while the effectiveness in terms of the partnership arrangements among organisations within the VSCFE sector, and the sustainability in terms of continuing this collaborative model of working beyond the funding period are described using qualitative findings.

### Setting

Luton is recognised as a super-diverse town characterised by a rich mixture of nationalities, ethnicities, religions, languages, and cultures. The population includes substantial communities from South Asia, Eastern Europe, Africa, and the Middle East, contributing to a vibrant and varied social fabric (23). In addition to this diversity, Luton experiences significant socio-economic deprivation, with higher rates of poverty, unemployment, and poor housing compared to national averages, factors strongly linked to health inequalities (24). The Marmot Review (2020) and subsequent updates emphasise that such social determinants: education, employment, housing, and community safety are critical drivers of health and wellbeing, and Luton’s profile reflects these wider structural challenges (25, 26). Hence, Luton was designated as the UK’s first Marmot Town in 2022 based on Luton’s health inequalities report (25), recognising its commitment to reducing health inequalities and improving social determinants of health through targeted local action (25–27). The Collaboration Fund had five lead organisations, and each of these lead organisations worked collaboratively with their partner organisations. Together, they addressed needs related to cost of living, food poverty, health, housing, employment, mental wellbeing, and community engagement through hubs, workshops, and specialist support.

### Data collection—impact

Five (*n* = 5) impact reports were submitted by all the lead organisations (in coordination with their partner organisations). These were included in the evaluation to measure the impact of the Collaboration Fund. Each report, provided in Excel format as required, contained quantitative data on service users, including gender, ethnicity, and postcode. No service usernames were provided, so beneficiaries could not be identified. Only consistently collected data across the five organisations were used in the evaluation.

### Data collection and participant recruitment—effectiveness and sustainability

The Collaboration Fund Project Coordinator at BLCF provided the contact information of project leads representing five lead organisations. Using these details, specific eligibility criteria guided the recruitment of project leads and project managers (including partner organisations) for semi-structured interviews. The evaluation used purposive sampling (28, 29), focusing on participants (i.e., stakeholders) who were responsible for overseeing and managing services funded by the Collaboration Fund. Individuals newly appointed to these roles who had not contributed to the delivery of services through the Fund were excluded.

Each was sent an information sheet and consent form via email. A total of four project leads (*n* = 4) and nine project managers (*n* = 9) returned signed consent forms, confirming their willingness to be contacted and participate in the evaluation. Multiple attempts were made to engage the remaining one project lead and four project managers. However, despite follow-up efforts, no affirmative responses were received from them (see Table 1). Semi-structured interviews were conducted using a separate topic guide for project leads and a separate topic guide for project managers from lead and partner organisations, respectively (Supplementary files 1 and 2).

**Table 1:** Sample of the final project lead (*n* = 4) and project managers (*n* = 9) from lead and partner organisations, respectively, who participated in the evaluation (20).

S. No	Staff number	Lead/Partner organisation	Gender	Ethnicity
1	Project Lead 1	Lead Organisation 2	Female	Black African
2	Project Lead 2	Lead Organisation 3	Female	White British
3	Project Lead 3	Lead Organisation 4	Female	White British
4	Project Lead 4	Lead Organisation 5	Female	South Asian
5	Project Manager 1	Partner Organisation 2.1	Female	White British
6	Project Manager 2	Partner Organisation 2.2	Male	South Asian
7	Project Manager 3	Partner Organisation 2.3	Female	White Irish
8	Project Manager 4	Partner Organisation 3.1	Female	White British
9	Project Manager 5	Partner Organisation 3.2	Female	South Asian
10	Project Manager 6	Partner Organisation 3.3	Female	South Asian
11	Project Manager 7	Partner Organisation 5.1	Male	South Asian
12	Project Manager 8	Partner Organisation 5.2	Female	White: Other
13	Project Manager 9	Partner Organisation 5.3	Female	South Asian

### Data analysis

Quantitative data from the impact report were analysed using descriptive statistics and are presented as frequencies and proportions. Postcode data, which informs the residential location of the service users, is presented in the form of a geospatial visualisation figure, enhancing the understanding of service reach, distribution, and accessibility (30). The maps are based on the Lower Layer Super Output Area (LSOA), a clustering method of geographic area used for statistical reporting, census, and other government datasets to present small-area statistics, such as population, health, economic, and social data (31). Qualitative data from 13 interview transcripts (*n* = 13) were exported into NVivo software version 20 (32), where they were coded and analysed using Framework Analysis (33). This included familiarisation, identifying a thematic framework, indexing, charting, mapping, and interpretation. Following this, findings are presented thematically in line with the aim of the evaluation—effectiveness and sustainability (33–35), providing a systematic and transparent method to organise and interpret qualitative data (33, 34).

### Findings

Findings are presented for impact, effectiveness and sustainability as per the aim of the evaluation.

### Impact

As outlined in the purpose of this evaluation, the impact of the Collaboration Fund was assessed in terms of short-term outcomes, specifically the reach of the programme. Quantitative findings from the impact report therefore, focused on indicators of programme reach, including the number of service users, gender distribution, new and returning service users, ethnicity of service users, and the geographical distribution of service users based on postcode.

**Figure 1:**
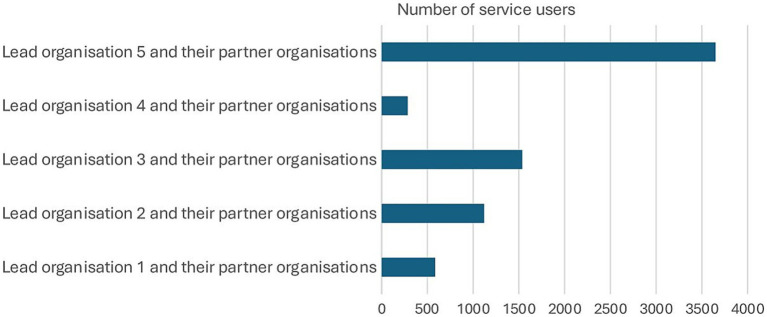
Total number of service users across the five lead organisations, along with their partner organisations. Adapted from “Collaboration Fund Evaluation Report Executive Summary May 2025” by Britzer Paul Vincent.

Through the Collaboration Fund, the lead and partner organisations engaged with a total of 7,177 service users. The breakdown of the number of service users from each lead organisation, along with their respective partner organisations, is illustrated in Figure 1 below.

**Figure 2:**
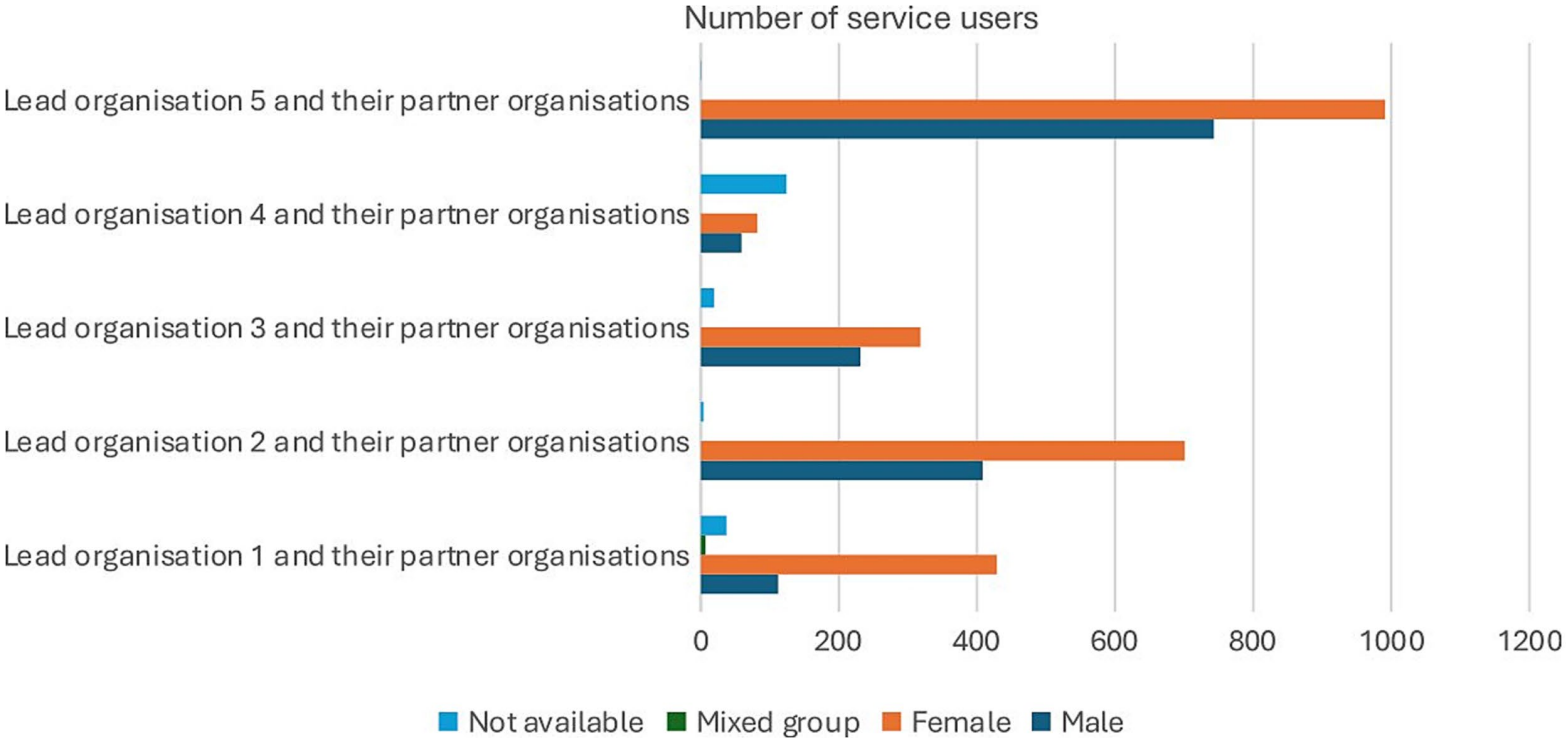
Breakdown of service users based on their gender. Adapted from “Collaboration Fund Evaluation Report Executive Summary May 2025” by Britzer Paul Vincent.

The majority of service users across all lead and partner organisations were female. Organisations also recorded a mixed category, where male and female service users were recorded as either a group or a family. As a result, the total number of service users is a reflection of families, groups and individuals. These discrepancies mean that the number of service users in Figure 2 will not add up to the total number of beneficiaries reported (7,177).

**Figure 3:**
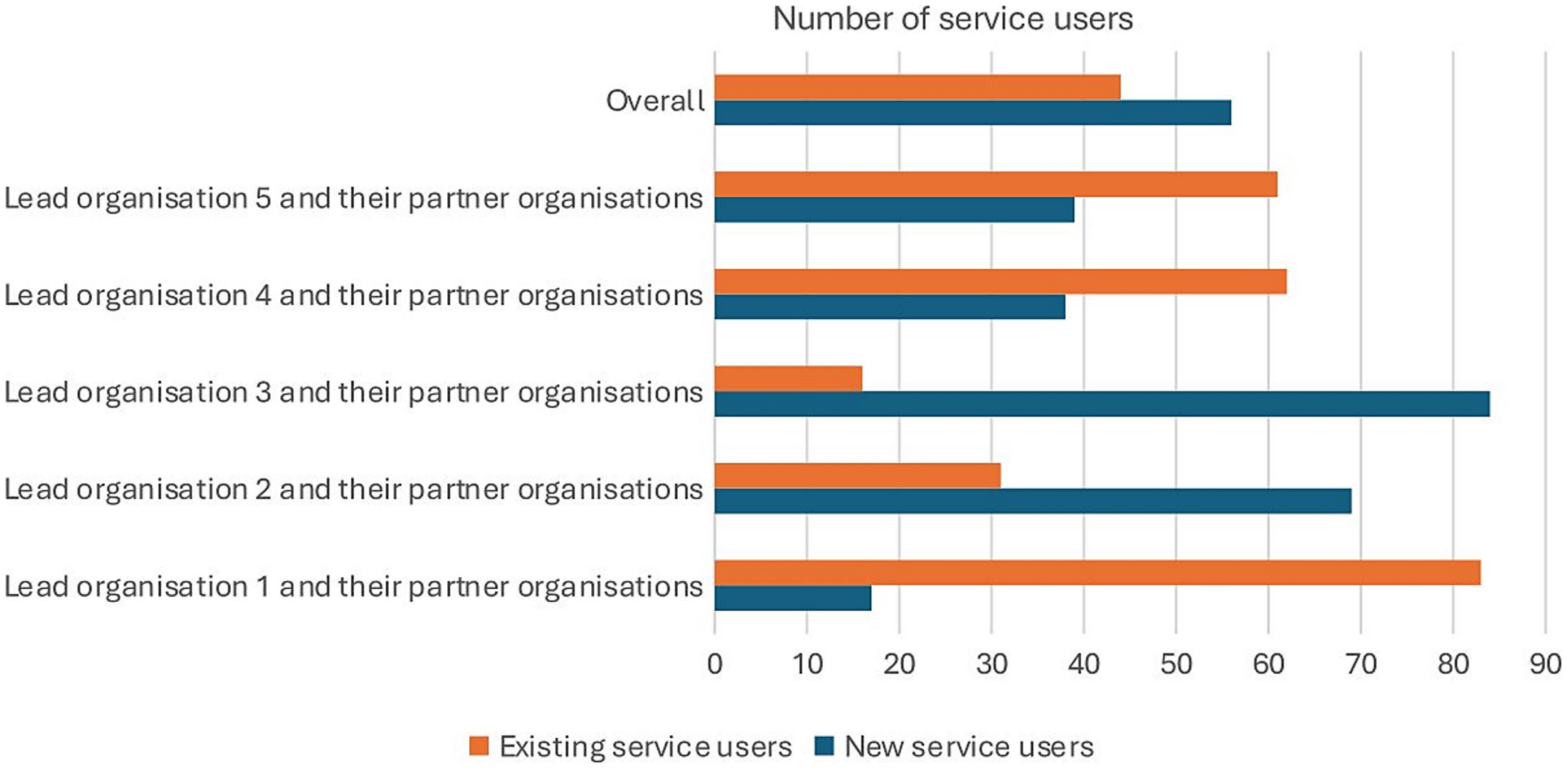
Breakdown of service users as new and existing service users. Adapted from “Collaboration Fund Evaluation Report Executive Summary May 2025” by Britzer Paul Vincent.

Overall, there was almost an equal number of new and existing service users. Figure 3 shows that there were differences in service users for each organisation and their partners. For example, Lead organisation 1 and its partner organisations primarily served existing service users, whereas Lead organisation 3 and its partners mainly engaged new service users (Figure 3).

**Figure 4:**
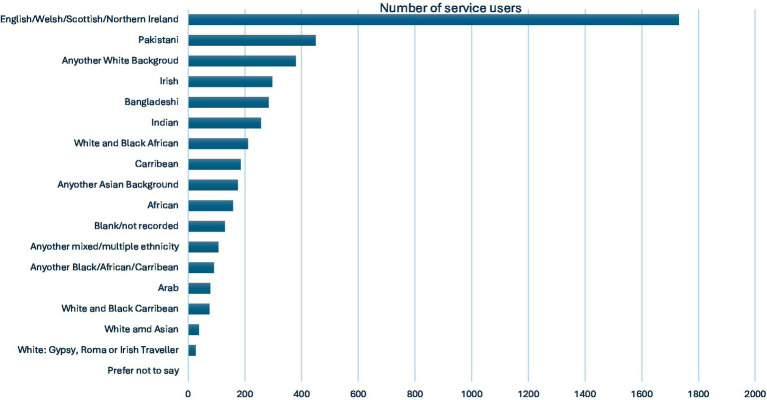
Overall service users by ethnicity. Adapted from “Collaboration Fund Evaluation Report Executive Summary May 2025” by Britzer Paul Vincent.

**Figure 5:**
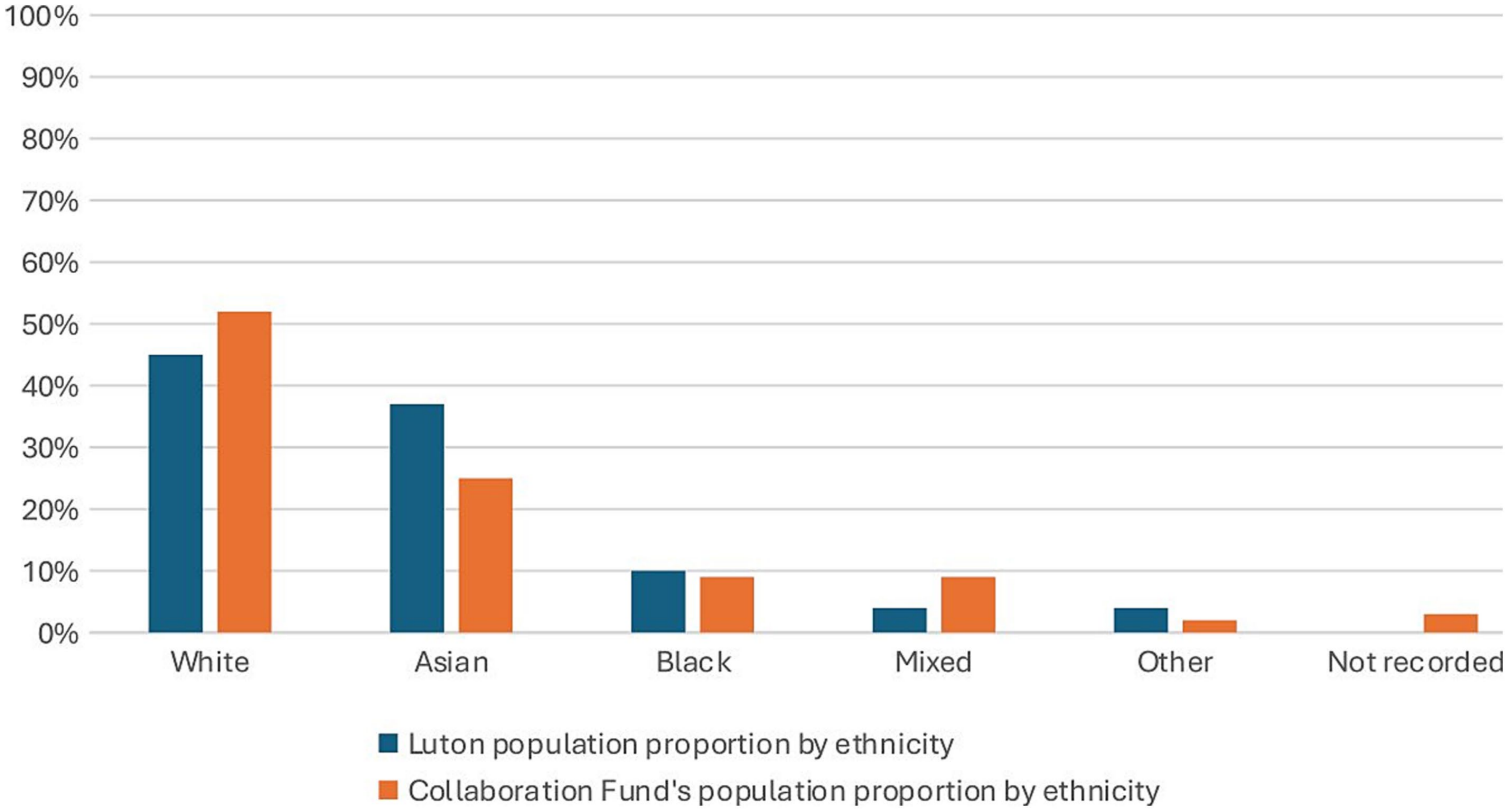
Comparison of Collaboration Fund beneficiaries’ ethnicity population proportion to Luton’s Ethnicity population proportion. Adapted from “Collaboration Fund” and “Luton’s Ethnicity population proportion” (Collaboration Fund Evaluation Report Executive Summary May 2025) by Britzer Paul Vincent.

Overall, the largest group of service users was identified as English/Welsh/Scottish/Northern Irish (52%, *n* = 1,730). The next largest group consisted of service users of Pakistani descent (9.64%, *n* = 450) (Figures 4, 5). The evaluation also showed that the distribution of ethnicity was varied across different organisations and their partners. Lead organisations 3, 4, and 5 and their partner organisations were able to deliver their services to a larger number of people from different ethnicities, when compared to other organisations. The reasons for this are detailed in the effectiveness section of the evaluation.

The evaluation showed that there was a marked underrepresentation of individuals from Black, Asian, and ethnic minority groups (Figure 5).

The evaluation found that the majority of service users were distributed in Central, South, Round Green, Farley, High Town, Vauxhall, Biscot, Beech Hill, and Dallow wards of Luton. These areas are identified as deprived (except for Round Green) according to the Index of Multiple Deprivation 2019.

**Figure 6:**
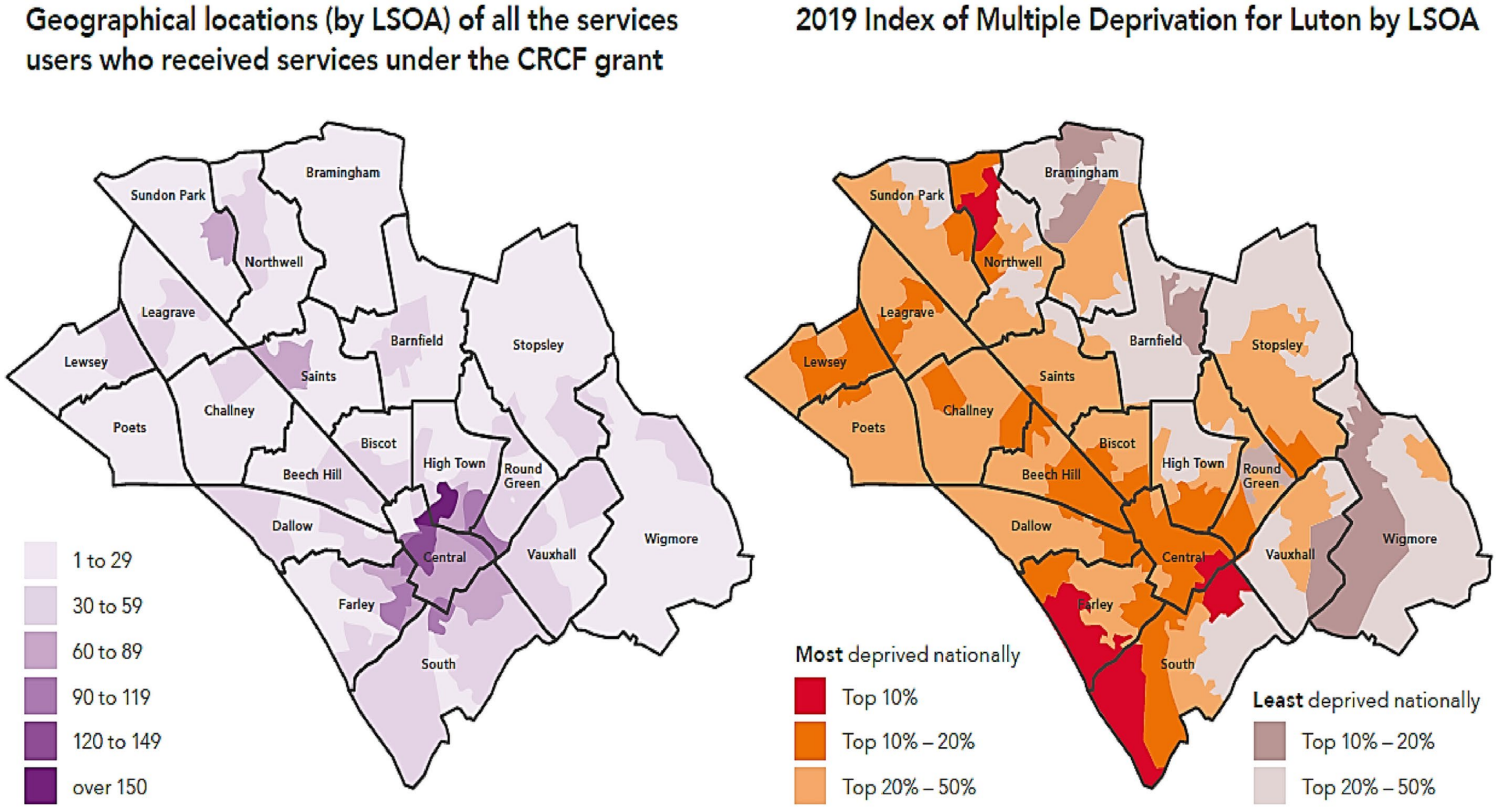
Geographical distribution of service users by LSOA and 2019 Index of Multiple Deprivation for Luton by LSOA. Reproduced from “Geographical distribution of service users by LSOA” and “2019 Index of Multiple Deprivation for Luton by LSOA” (Collaboration Fund Evaluation Report Executive Summary May 2025) by Britzer Paul Vincent.

This shows an association between beneficiaries living in deprived areas and access to organisations and their partners (Figure 6).

### Effectiveness

This section focuses on the effectiveness of the services provided through the Collaboration Fund, in terms of the partnership arrangements among organisations within the VSCFE sector. It focuses specifically on the positive impact of working collaboratively, challenges in working collaboratively, facilitators to community engagement, and the effect of partnership functioning on the inclusivity of service provision. Inclusivity is presented as part of the effectiveness findings because it reflects not the scale of engagement in numerical terms, but rather the extent to which partnership arrangements effectively enabled equitable access to services.

#### The positive impact of working collaboratively

Working collaboratively allowed the lead and partner organisations to utilise their diverse skills and expertise, making the partnership more productive. The format gave organisations and their partners time and capacity to co-design the services by drawing on their diverse expertise. This made a significant impact on the way they worked and delivered their services. It helped the project leads and managers widen their network and strengthen the partnership. This was identified as one of the strengths of working collaboratively. A project manager said:

Bringing everybody together, we were able to brainstorm different ideas… gave us ideas how we could… get support from other organisations, how they can help us… One of the strengths and benefits I think of working together [is]… there was real strength in that partnership… what it's done for me and the organisation is that it has opened a network (Project Manager 9, Partner Organisation 5.3).

One of the project leads noted that, although they had previously worked with other VCSFE organisations in the community, the Collaboration Fund transformed how they collaborated with them:

But having that time and space to think out-of-the-box and make those connections and maybe even have time to think… With the collaboration fund, it got all of us together… where we were then able to brainstorm ideas, implement them. Everybody took responsibility… So sometimes when you're working on your own, you know it's just your ideas, isn't it? But when there's four professional heads working on a project, there's lots of different ideas. It benefits the community (Project Lead 4, Lead Organisation 5).

Stakeholder highlighted that bringing lead and partner organisations together in outreach activities allowed service users to access multiple services in one location (e.g., community halls, churches, community hubs and warm hubs). This approach to service delivery helped address previous challenges, where, before the Collaboration Fund, service users had to visit several locations and organisations to access different services. Working together enabled the sharing of resources among the lead and partner organisations, including venue spaces for delivering services that required private rooms. One of the stakeholders mentioned:

But where the collaboration occurred, it was bringing other partners in physically on the same day. So that clients could see more than one type of service and more than one type of specialist advice [at the outreach]… Collaborations could be strengthened further by incorporating more resource sharing… when [partner organisations] lacked private rooms, [lead organisation] were able to provide a private and confidential space to meet with clients. Future opportunities for this could be explored. Particularly where there is a strong crossover of clients supported (Project Lead 1, Lead Organisation 2).

#### Challenges with working collaboratively

The overlap of the services provided by the lead and their respective partner organisations limited them from working collaboratively, particularly with referrals of clients across them. This was because the lead organisation may not have seen the need to refer service users, given that they are already providing the service requested or needed by the user. One of the project managers noted:

I love collaboration. I think it's a really good idea… And I think it could be really good if the organisations that work together were very different… The problem here is… I think maybe we crossed over a little bit too much… So, I think… the lead partnership… they do great stuff, but should they be partnered with someone else, who does similar things in a collaboration, I think probably not (Project Manager 1, Partner Organisation 2.1).

Partner managers highlighted that the project lead was often the mediator communicating with Funders. While the lead organisation benefitted from its relationship with the funders, the indirect communication approach led to a perceived loss of power and influence, as partners were unable to engage with the Funders directly. A project manager reported:

We were informed by the lead organisation, but we weren't directly benefiting from the funder themselves because their communication was with the lead organisation (Project Manager 5, Partner Organisation 3.2).

#### Facilitators for community engagement

Stakeholders highlighted that higher levels of engagement occurred when they had an established and trusted relationship with the public. In contexts where these organisations possessed a strong reputation and were recognised for delivering high-quality services, they were better positioned to cultivate meaningful engagement. As a result of this, their services were mainly known in the community through word of mouth. One of the project leads said:

I think it is an advantage for us, because we're not associated with the [local authority], we're not associated with any legal body or anything. We're a charity, so people find it quite… accessible… And I think like I said to you before, because we're giving them something tangible… it builds that trust with people… Rather than someone not giving you anything and always questioning (Project Lead 4, Lead Organisation 5)

However, in addition to this, engaging with the media improved the sharing of information on the services provided by the lead and partner organisations. Sharing videos and other effective media approaches increased the usage of services. A project manager stated:

The best way to promote this, would be to do show little videos, so we did show little videos to promote the actual activities before they took place… We did a Facebook and YouTube live broadcast of the actual workshop itself, so others who are watching online can also benefit from the information given (Project Manager 7, Partner Organisation 5.1)

#### Effect of partnership functioning on the inclusivity of service provision

A key challenge when receiving help through outreach was the stigma. A project lead mentioned that hesitancy to seek help among service users arose mainly from concerns that others might view them as struggling or incapable of managing their daily needs. In response, a carefully designed plan combining creative activities with the services provided promoted inclusion and helped reduce the stigma associated with receiving support. A project lead mentioned:

We did another event that was… actually, we made that all about sort of gaming and called it gaming and school supplies. And that was fully booked, and loads of people came… And I think one of the most successful things we did was… doing a dance and dinner programme. And that was… doing a dance workshop and then providing a free meal afterwards (Project Lead 3, Lead Organisation 4)

Employing staff and volunteers from diverse backgrounds (ethnicity, religion, and culture), reflecting the demographics of the Luton community, helped make services feel more inclusive for the service users. The project leads and managers highlighted that the multilingual background of staff enabled them to overcome the language barriers, further enhancing inclusivity within services. Those who did not have a diverse workforce relied on external interpretation of services. This proved challenging for smaller partner organisations. A project lead highlighted this as follows:

If we haven't got someone who speaks their language, we use something called Language Line. So, we'll actually phone through and get an interpreter. But that costs money. So, what we try and do is make sure our volunteers and our staff are representative of the demographic in Luton. So, we've got a lot of community language speakers in here. So, we try and do that (Project Lead 2, Lead Organisation 3).

### Sustainability

Lead and partner organisations reported that maintaining the same level of partnership activity, achieved during the Collaboration Fund period, was reported to be difficult within the allocated grant envelope. Project leads and managers highlighted that continuing services, such as outreach, involved expenses, such as venue hire and travel. This was identified as a barrier to sustaining the work initiated under the Collaboration Fund. To address this, stakeholders and project managers recommended enhancing opportunities for ongoing collaboration. They suggested strengthening networks within Luton’s existing VCSFE sector and establishing a shared platform for organisations to co-host outreach events using existing resources. They also proposed increasing access to funding opportunities that specifically support collaborative initiatives. A project manager mentioned:

They [other VCSFE organisations in Luton which are not a part of the Collaboration Fund] refer to us… because they know that we're doing arts and crafts for adults. So definitely, referrals could work… So recently we've started to work in the (XXX) ward with [another organisation]… So, they’re thinking, hang on, you guys are possibly a little bit more experienced than us… So, I think working together definitely helps (Project Manager 9, Partner Organisations 5.3).

Project leads and project managers acknowledged that the Fairness Taskforce allowed them to widen their networks with various VCSFE organisations, and they highly appreciated. The Fairness Taskforce in Luton, UK, is a community-based group that works to address inequality and structural barriers. It aims to create a more equitable society for all residents. However, project leads and project managers highlighted that attending the Fairness Taskforce meetings was taking resources away, given that some of the VCSFE organisations are smaller and need all hands to deliver service and find it difficult to attend such impactful and significant events. Alternatively, they suggested creating a directory with a 50-word summary of each VCSFE sector in Luton, outlining their services. A project lead reported:

I have to manage the charity. I don't have time to go to meetings all the time… And in that meeting, I can't speak to everybody because there are too many people… You know the VCSFEs should have a small update, maybe 50 words that everybody can then read and check what's what everybody's doing (Project Lead 4, Lead Organisation 5).

## Discussion

The Collaboration Fund was established as a strategic continuation of post-COVID recovery efforts in Luton, building on previous funding streams to strengthen collaboration within the VCSFE sector. Operating between January 2023 and June 2024, the Collaboration Fund aimed to address health inequalities by supporting partnerships that deliver integrated, community-based interventions to marginalised and clinically vulnerable populations. Recognising the unique role of VCSFE organisations in reaching underserved groups and addressing wider determinants of health, the fund brought together five lead organisations and their partners to co-produce locally tailored services. This discussion critically reflects on the impact, effectiveness, and sustainability of the collaborative dynamics of the funded activities, drawing from the evaluation findings. The short-term objectives of the Collaboration Fund were to assess the reach of services delivered through the collaborative model of working, evaluate the effectiveness of partnerships among organisations within the VSCFE sector, and explore the sustainability of this collaborative approach beyond the funding period. In the longer term, the initiative aimed to improve the overall wellbeing of vulnerable populations and contribute to reducing health inequalities.

The Collaboration Fund benefited 7,177 service users across a range of services, including mental health, housing, financial and social support, and mobility assistance. It fostered collaboration among community organisations, enabling collective rather than competitive working, which allowed service users to access multiple resources in the outreach events and enhanced the efficiency and comprehensiveness of support. These findings demonstrate that leveraging the VCSFE sector collaboratively adds value for money for both service users and funding. The challenges identified through this evaluation inform future strategies to optimise collaborative working models and maximise impact.

The evaluation found that the majority of service users across lead and partner organisations were female, reflecting wider trends in the VCSFE sector where women are often the primary users of services (35). However, even though the Collaboration Fund offered services relevant to everyone and scheduled them at various times, including daytime, evenings, and weekends, to accommodate those balancing work and caring responsibilities, male engagement still remained low. This highlights the need for further investigation into the barriers and facilitators influencing males’ engagement with, and receipt of support from, the VCSFE sector. Potential contributing factors may include traditional masculine norms, stigma associated with males seeking help, limited male representation within services, and logistical challenges related to service delivery (36–38).

The evaluation of services provided through the Collaboration Fund revealed a complex interplay of challenges and enablers that shaped the overall effectiveness of delivery. One of the key concerns was the underrepresentation of Black, Asian, and minority ethnic service users, echoing findings in national studies (39). Despite Luton’s super-diverse demographic, the data showed a mismatch between the population profile and those accessing services. Given Luton’s ethnic diversity and health inequalities (40), this raises concerns about the inclusivity of service delivery. Barriers cited include language, cultural stigma, and limited outreach. The findings reflected a lack of inclusivity within service delivery, demonstrating the need for organisations to ensure adequate representation and strengthen their cultural awareness. Future interventions should therefore be culturally tailored to enhance accessibility (41, 42).

This prompted reflections among project leads and managers, many of whom pointed to the enduring stigma attached to help-seeking within some communities. Fears of judgment or cultural taboos around mental health and social support were significant barriers, concerns that align with national studies documenting similar experiences across the UK (43, 44).

Some services responded to this challenge by embedding creative engagement methods into their outreach strategies. These included dance, arts, and family-based activities that subtly normalised the act of seeking support, drawing participants into services through more socially acceptable means. Such approaches resonate with findings from other works (45, 46), which highlight the value of the arts in reducing stigma and promoting early help-seeking. The potential of creativity to act as both an engagement tool and a therapeutic medium was recognised by the project leads and managers.

Language and communication barriers further compounded issues of access. In a linguistically diverse town, several services benefited from having staff and volunteers who spoke community languages, improving trust and comfort for non-English speakers. This practical solution was considered more sustainable than relying solely on interpretation services, which, although effective, were seen as costly and inconsistent in quality (47). The use of mobile translation apps was also mentioned by the participants as a suggested tool to help with translation. A study that examined the use of mobile translation applications found that, while these tools can support learning and enhance performance in straightforward tasks, their reliability in nuanced service contexts remains uncertain. Specifically, mobile apps may struggle with context-specific terminology, idiomatic expressions, and cultural subtleties, limiting their suitability for professional or sensitive interactions (48).

Some stakeholders raised concerns over blurred roles and overlapping responsibilities, which in certain instances led to duplication or inhibited innovation within service delivery and partnerships. Having multiple team members with the same skills can make an organisation more resilient, but if roles are not clearly defined, it can cause confusion, lower efficiency, and hinder effective teamwork. A study highlighted that in a cross-sector collaboration in public service delivery, overlapping of responsibilities among partner organisations initially led to duplicated efforts and delays in decision-making (49). Similarly, achieving collaborative advantage in such a situation depends on balancing complementary skills with clarity in responsibilities to foster trust, coordination, and joint problem-solving (50).

Findings from the interviews suggest that while the Collaboration Fund enabled strong multi-agency working, sustaining this momentum post-funding has proven difficult. Project leads and managers consistently pointed to financial constraints as a major barrier, with outreach activities requiring venue hire and travel costs that smaller organisations are often unable to absorb. The integrated outreach model, where service users accessed multiple services in one location, was praised for its efficiency. However, in the absence of funding, organisations reverted to cross-referrals that required service users to travel between sites, reducing ease of access and continuity of care.

Despite these constraints, some organisations adopted more sustainable forms of collaboration by participating in each other’s community events and co-hosting outreach activities. Partnering with trusted organisations in the community was seen as particularly effective in enhancing visibility and reach, an approach also validated by evidence from similar initiatives elsewhere (51). However, concerns were raised due to limited funding streams that support and prioritise collaborative approaches, which risks fragmenting services and weakening partnerships built.

The need for ongoing, practical networking opportunities was a recurring theme. Initiatives like the Fairness Taskforce (52) were highly appreciated for fostering cross-sector understanding; some managers noted the time burden of attending regular meetings, especially for smaller organisations with limited staff. A proposed solution was to develop a more inclusive, co-produced VCSFE directory. However, previous research has shown that their effectiveness is often compromised by top-down control, excluding newer or smaller organisations (53, 54). To avoid this, future tools must be co-created with the sector and regularly updated to reflect its diversity and dynamics. Without such inclusive approaches, efforts to sustain collaboration risk becoming superficial and unsustainable. It is also crucial to consider that such approaches also have a financial implication.

This evaluation used a mixed methods approach, enhancing rigour by providing a more comprehensive understanding of the Collaboration Fund. The integration of diverse methods and perspectives strengthened the findings, and the involvement of an independent evaluation team ensured neutrality and created a safe space for honest reflective contributions. Earlier involvement of the evaluation from the beginning would have allowed for better data collection, including improving recruitment of key personnel. Some of the stakeholders had left or changed roles, making it harder to reach out for their participation in the evaluation. Despite these challenges, the evaluation produced valuable insights to guide future collaborative funding and evaluation practices.

## Conclusion

In the COVID recovery period, Collaboration Fund enabled meaningful partnerships among VCSFE organisations in Luton, leading to more inclusive, community-based service delivery that addressed the needs of groups disproportionately affected by the pandemic. By facilitating multilingual outreach, enhancing staff capacity, and supporting creative and social engagement, the fund contributed to improved access, reduced isolation, and greater community participation. However, challenges around sustainability, accessibility, and resource demands highlight the need for continued investment and strategic support to maintain and build on these gains in the long term. To improve access among Black, Asian, and Minority Ethnic populations, the VCSFE sector should employ staff reflective of the communities they serve. Effective collaboration can be achieved when less diverse organisations partner with those that have greater community representation, and by adding creative components to service delivery.

## Data Availability

The original contributions presented in the study are included in the article/supplementary material, further inquiries can be directed to the corresponding author.
